# Increased Alcohol Consumption in Mice Lacking Sodium Bicarbonate Transporter NBCn1

**DOI:** 10.1038/s41598-020-67291-0

**Published:** 2020-07-03

**Authors:** Jesse R. Schank, Soojung Lee, Carlos E. Gonzalez-Islas, Sadie E. Nennig, Hannah D. Fulenwider, Jianjun Chang, Jun Ming Li, Yejin Kim, Lauren A. Jeffers, Jaegwon Chung, Jae-Kyung Lee, Zhe Jin, Christian Aalkjaer, Ebbe Boedtkjer, Inyeong Choi

**Affiliations:** 1 Department of Physiology and Pharmacology, University of Georgia College of Veterinary Medicine, Athens, GA 30602 USA; 2 Department of Physiology, Emory University School of Medicine, Atlanta, GA 30322 USA; 3 Department of Medicine, Pulmonary Division, Emory University School of Medicine, Atlanta, GA 30322 USA; 4 Department of Neuroscience, Uppsala University, Uppsala, 75124 Sweden; 5 Department of Biomedicine, Aarhus University, 8000, Aarhus C, Denmark

**Keywords:** Transport carrier, Addiction

## Abstract

The previous reports on an addiction vulnerability marker in the human SLC4A7 gene encoding the Na/HCO_3_ transporter NBCn1 suggest that this pH-regulating protein may affect alcohol-related behavior and response. Here, we examined alcohol consumption and sensitivity to the sedative effects of alcohol in male NBCn1 knockout mice. These mice displayed lower pH in neurons than wildtype controls, determined by intracellular pH in hippocampal neuronal cultures. Neurons from knockout mice had a higher action potential threshold and a more depolarized membrane potential, thus reducing membrane excitability. In a two-bottle free choice procedure, knockout mice consumed more alcohol than controls and consistently increased alcohol consumption after repeated alcohol deprivation periods. Quinine and sucrose preference was similar between genotypes. Knockout mice showed increased propensity for alcohol-induced conditioned place preference. In loss of righting reflex assessment, knockout mice revealed increased sensitivity to alcohol-induced sedation and developed tolerance to the sedation after repeated alcohol administrations. Furthermore, chronic alcohol consumption caused NBCn1 downregulation in the hippocampus and striatum of mice and humans. These results demonstrate an important role of NBCn1 in regulation of alcohol consumption and sensitivity to alcohol-induced sedation.

## Introduction

pH is a broad but important biological factor that can cause significant changes in brain function^1,2^. pH can change activities of numerous proteins such as neurotransmitter receptors, ion channels, and synaptic transmission machinery proteins, and such changes perturb membrane excitability, firing properties, and intracellular signaling cascades^3,4^. The physiological importance of pH in neurons has been documented^5,6^; nonetheless, it is presently unclear whether pH affects reward, motivation and addiction. In the field of addiction research, pH disturbance has been viewed as the consequence of drug action or metabolism^7^. For example, the risk of metabolic acidosis is increased by an overdose of drugs including cocaine, heroin, and ecstasy^8^. Metabolic acidosis is also induced by chronic or heavy alcohol consumption^9,10^. This metabolic complication is due to the conversion of alcohol to ketone bodies such as β-hydroxybutyrate, thus consuming HCO_3_^−^. For example, a study shows that about 25% of alcoholic admissions in hospitals have ketogenic acidosis^11^, which affects many organ functions. Nonetheless, little is known whether pH conversely affects drug consumption or drug-induced response.

NBCn1 (SLC4A7) is a pH-regulating plasma membrane protein found in a variety of tissues^12^. In most neurons, NBCn1 is predominantly localized to postsynaptic membranes^13^, where it transports Na^+^ and HCO_3_^−^ into neurons and buffers intracellular H^+^. Thus, the transporter regulates intracellular pH (pH_i_) in postsynaptic neurons and extracellular pH (pH_o_) in the synaptic cleft. Genome-wide association studies discovered the single nucleotide polymorphism rs3278, in the human SLC4A7 gene, prevalent among abusers who used cannabinoid, cocaine, heroin, or nicotine in the US population^14^. This polymorphism is also prevalent in alcohol abusers in the Collaborative Studies on Genetics of Alcoholism pedigrees and in substance abusers who displayed the maximum possible 3+ on the drug use scale^15^. Recently, we reported that the genomic function of rs3278 is to enhance an alternative gene transcription for an N-terminally deleted protein that has a defect in membrane expression and alters pH_i_^16^. This finding suggests that abnormal function in NBCn1 is likely a physiological indicator of substance abuse or may directly affect addiction vulnerability.

In this study, we investigated alcohol-related behaviors in NBCn1 knockout (KO) mice to test whether NBCn1 loss alters alcohol consumption. First, we examined the effects of NBCn1 loss on pH_i_ recovery from acidification and resting pH_i_, as well as membrane excitability, in hippocampal neurons from KO and wildtype (WT) mice. Hippocampal neurons were chosen because the cellular and functional properties of NBCn1 in these neurons have been determined^13,17,18^, and such information should help us to investigate the mechanism by which NBCn1 influences alcohol’s effects on neurons^19^. We then examined alcohol-related behaviors in KO mice to determine voluntary alcohol consumption, sweet and bitter taste sensitivity, reward potency of alcohol, and sensitivity to alcohol-induced sedation. In addition, a set of experiments were performed to test whether NBCn1 levels were affected by chronic alcohol consumption. The results show that NBCn1 affects alcohol consumption and sensitivity to alcohol-induced sedation in mice. Given that a small pH difference has been considered minor in function, a marked change in alcohol-related behaviors by NBCn1 loss is intriguing.

## Material and Methods

### Mice

The experiments in this study were conducted in accordance with the National Institute of Health Guide for the Care and Use of Laboratory Animals and were approved by the Institutional Animal Care and Use Committee at both Emory University and University of Georgia. All experiments in this study were performed with male mice to minimize a potential gender difference. The generation and basic characterization of NBCn1 KO mice on a C57BL/6 background, including genotyping and breeding strategies, were described previously^20^. Other physiological properties were characterized previously^21,22^. Heterozygotes were bred to generate KO mice and WT littermates, and genotyping was done by PCR of tail DNA. WT littermates served as controls for pH_i_ measurements and electrophysiological recordings. WT littermates and age-matched C57BL/6 mice (Jackson Labs; Bar Harbor, ME, USA) consumed alcohol similarly and thus C57BL/6 mice were used in alcohol-drinking tests. Mice were housed on a 12 h light/dark cycle and experiments were conducted during the light phase. Mice were provided with standard chow and water *ad libitum*.

### Measurements of pH_i_ in hippocampal neuronal cultures

Primary cultures of hippocampal neurons were prepared from P1–P7 postnatal mice using the protocol^23^ with slight modification. Briefly, mouse brains were removed after decapitation and placed in Eagle’s balanced salt solution containing 10 mm HEPES and 1 mm sodium pyruvate. Hippocampi were removed and digested with papain (Worthington Biochemical Corporation; Lakewood, NJ, USA) for 15 min at 37 °C, and then triturated with a sterile pasteur pipet. Dissociated neurons were then plated on poly-L-lysine-coated coverslips at a density of 4 × 10^5^ cells in a 60-mm petri dish. Neurons were plated with Neurobasal/B27 medium and incubated in a 5% CO_2_ humidified chamber at 37 °C. Cell viability, determined by lactate dehydrogenase release cytotoxicity assay, shows ~17% cytotoxicity in neurons from P5-P7 mice incubated for 7–10 days, thus validating the use of neuronal cultures (Supplementary Table S4). For pH_i_ measurements, neurons on a coverslip were loaded with 6.5 *μ*M of 2,7-bis(2-carboxyethyl)-5(6)-carboxyfluorescein acetoxymethyl ester (BCECF-AM) for 10 min and mounted in a closed perfusion chamber attached on the stage of a Zeiss Axiovert inverted microscope. The dye was alternately excited with 440 and 490 nm lights using a Lambda LB-LS Xenon Arc lamp (Sutter Instruments; Novato, CA), and the 535 nm emission lights from both excitations (i.e., *I*_490_ and *I*_440_) were captured using a Nikon NIS Elements AR 3.0 imaging software (Nikon; Melville, NY). The *I*_490_/*I*_440_ ratio was calculated after subtracting background from an area where there was no neuron. Dye calibration was accomplished by the nigericin method^24^. The chamber was perfused with HEPES-buffered solution (mM: 140 NaCl, 1 KCl, 1 MgSO_4_, 2 CaCl_2_, 2.5 NaH_2_PO_4_, 5.5 glucose, 10 HEPES, pH 7.5) and then applied with solution containing 5% CO_2_, 33 mM HCO_3_^−^ (NaHCO_3_ replaced NaCl and HEPES). Solutions also contained 10 μM of ethyl isopropyl amiloride to block endogenous Na/H exchangers in neurons. The rate of pH_i_ recovery (dpH_i_/dt; pH_i_ change per sec) was calculated using the slope during the first 2 min of recovery from a CO_2_-induced acidification.

### Whole cell patch clamp

Whole-cell recordings in a current clamp configuration were performed from hippocampal neuronal cultures 7–10 days after plating. The amplifier bridge circuit was adjusted to compensate for electrode resistance and was subsequently monitored. A series of currents (20 pA increments) were applied to determine the first current step capable of eliciting one action potential. Recordings were performed in a solution that contained (mM): 124 NaCl, 3 KCl, 2 CaCl_2_, 1 MgCl_2_, 1.25 NaH_2_PO_4_, 26 NaHCO_3_, 10 glucose and was saturated with 95% O_2_, 5% CO_2_. The patch pipette contained 10 NaCl, 36 KCl, 94 K-gluconate, 0.1 CaCl_2_, 1 MgCl_2_, 10 HEPES, 1.1 EGTA, 1 Na-ATP, and 0.1 Mg-GTP. pH was adjusted to 7.2 and osmolarity was adjusted to 290 ± 1 mOsm. Recordings were done using an Axopatch 200B amplifier controlled by pClamp 10. Resting membrane potentials were recorded under I = 0 mode. Experiments were conducted at room temperature.

### Two-bottle free choice alcohol drinking

Two-bottle free choice was performed as described previously^25^. Briefly, mice were individually housed and allowed continuous access to two bottles: one bottle containing water and a second bottle containing alcohol with gradually increasing concentrations of 3, 6, 9, 12, and 15% alcohol (v/v). Each concentration was available for 4 days before being increased by 3%. Every time the bottles were weighed, they were switched to minimize a potential side preference. Drinking was measured for an additional 2 weeks until the baseline reached a stable level (defined as alcohol consumption that varied by <15% over three consecutive days). Repeated cycles of alcohol access and deprivation were carried out as described^26^. Briefly, after a stable baseline was achieved, mice were given 4 cycles of 6-day deprivations (both bottles contained water) followed by 1 day access to alcohol and water in the two-bottle choice procedure. Water and alcohol consumption was measured as g/kg/24 h, and alcohol preference (ratio of alcohol to total fluid intake) was determined.

### Quinine and sucrose preference

Quinine preference was assessed as described previously^25^. A separate cohort of mice was individually housed and presented with two bottles in the home cage. Both bottles contained water and were weighed every 24 hours. Bottles were switched daily. Once the baseline water consumption reached stability, 0.001–0.3 mM of quinine hydrochloride was added to one of the water bottles. Each quinine concentration was offered for 1 day; in between each quinine test day, animals were given two bottles of water for 2 days. For sucrose preference, 1% or 10% (w/v) of sucrose and water were offered for 2 days in two bottles. Quinine or sucrose preference was determined as a ratio of taste solution intake to total fluid intake.

### Loss of righting reflex (LORR)

Mice were intraperitoneally injected with 3.0 and 3.5 g/kg of alcohol, placed in a supine position on a V-shaped trough and assessed for inability to right themselves twice within a 30 sec period. Latency (time to onset of LORR) and duration (time elapsed between the onset of sedation and the recovery from LORR) were recorded. Recovery was considered when a mouse regained the ability to place all four limbs on the table surface twice within a 30 sec period. In a second cohort of mice, animals were injected with 2.0 g/kg of alcohol for 7 days and then with 3.0 and 3.5 g/kg of alcohol on day 8. Blood alcohol concentration (BAC) was determined by injecting 2 g/kg of alcohol and collecting blood samples from the submandibular vein at 5, 60, and 180 min post injection. BAC was determined using an Alcohol Assay Kit (Cell Biolabs, San Diego, CA, USA).

### Conditioned place preference (CPP)

Rewarding effects of alcohol were assessed using CPP as previously described^27^. Briefly, the CPP chambers had two compartments: one had white walls/grid flooring and the other had black walls/bar flooring (Med Associates, Fairfax, VT, USA). The compartments were equipped with photocell beams that automatically measured activity and time on each side. Mice were habituated to the testing room for 1 hour before testing. During pretest, mice were given full access to the chamber for 15 min. The compartment in which mice were initially placed was random and alternated. Following the pretest, 5-min conditioning sessions were conducted daily for 3 days, with saline injections in the morning and 2 g/kg alcohol injections 4 hours later. Conditioning was performed using an unbiased and counterbalanced design, in which half of the animals received alcohol on their preferred side and half on their non-preferred side, and half of the animals received alcohol on the black side and half on the white side. On test day, mice were placed into the chamber without pretreatment and given free access to the chamber for 15 min. Preference scores were calculated by subtracting time spent on the saline paired side from time spent on the alcohol paired side. Locomotor activity was assessed as horizontal activity counts during each session.

### Immunohistochemistry

Brain tissues of chronically alcohol-fed mice and pair-fed mice were provided by Dr. Michael Koval (Department of Medicine, Emory). The procedure for alcohol feeding is described previously^28^. Briefly, alcohol was administered by increasing concentrations from 0% to 20% in 5% increments over a two-week period; mice were then maintained at 20% alcohol for an additional six weeks. Brains were fixed in 4% paraformaldehyde, dehydrated in 30% sucrose, and embedded in paraffin. Paraffin sections (5 μm thick) were heated, deparaffinized in xylene, rehydrated through graded alcohol rinses, and incubated in 3% H_2_O_2_ for 20 min. Antigen retrieval was done by heating in 10 mM citrate buffer (pH 4.0) in a microwave for 2 min. Sections were blocked with 1% bovine serum albumin in phosphate-buffered saline (PBS) and then with an anti-NBCn1 antibody^29^ diluted at 1:50 at 4 °C overnight. Sections were washed and incubated with an anti-rabbit horseradish peroxidase-conjugated secondary antibody (cat. #: 7074; Cell Signaling Technology, USA) diluted at 1:1000 for 1 hr at room temperature. Sections were stained with 3,3′-diaminobenzidine (DAB) and then counterstained with hematoxylin. Images were visualized using a NanoZoomer Scanner (Hamamatsu Photonics, Hamamatsu City, Japan). Full scanned images were evaluated with NPD.view2 software (Hamamatsu). Quantification of NBCn1 staining was done using ImageJ software (NIH, Bethesda, MD, USA) with color deconvolution to separate the hematoxilin and DAB stains^30^. DAA images (×20) were used. A threshold level to distinguish staining intensity from nonspecific background was adjusted using histograms and remained constant for all images to validate comparisons. DAB staining intensity was determined by positioning boxes around cell bodies at different locations on the image. An area where there was no neuron served as a background.

### Postmortem human dorsal striatum and qPCR

The experiments with postmortem human hippocampus (dentate gyrus) and dorsal striatum (putamen) were conducted in accordance with the ethical guidelines and practice approved by Uppsala University. The tissues were obtained from the New South Wales Tissue Resource Center, University of Sydney, Australia (http://sydney.edu.au/medicine/pathology/trc/index.php), where postmortem tissues from Caucasian males were collected by qualified pathologists under full ethical clearance. The full description of the brain bank, protocols, ethical approval and funding is described in Sheedy *et al*.^31^. Alcoholic tissues were obtained from individuals with alcoholism defined by the Diagnostic and Statistical Manual for Mental Disorders and National Health and Medical Research Council/World Health Organization. Control tissues were from individuals who had no alcohol or consumed ≤20 g of alcohol per day on average. Both alcoholics and controls were matched in age, post-mortem interval (PMI), brain pH, RNA quality indicator (RQI), and smoking history. The detailed demographic data for the subjects in the current study are listed in Supplementary Tables S1 and S2. Total RNA was isolated and quantified using Nanodrop (Nanodrop Technologies; Wilmington, DE, USA). RQI was measured using Bio-Rad Experion (Bio-Rad; Hercules, CA, USA) with Eukaryote Total RNA StdSens assay following the manufacturer’s manual. All RNA samples had >5 RQI values (average RQI of the samples was 7.5 ± 0.35 for controls and 7.5 ± 0.35 for AUD), which are generally considered high quality of RNA suitable for qPCR^32^. Reactions with human NBCn1-specific primers (Supplementary Table S3) were conducted at 95 °C for 5 min for an initial denaturation, and 45 cycles at 95 °C for 15 sec, 60 °C for 30 sec, and 72 °C for 30 sec. References β-actin (stability value M = 0.44), TATA-box binding protein TBP (M = 0.41), and ubiquitin C UBC1 (M = 0.41) were used for normalization according to the previously developed approach^33,34^.

### Statistical analysis

Data were reported as mean ± standard error of the mean. For statistical significance test, unpaired, two-tailed Student t-test was used for comparison between WT and KO mice of LORR, dpH/dt, pH_i_, CSF pH, and patch-clamp recording parameters. The unpaired, two-tailed Student t-test was also used for comparison of NBCn1 levels in pair-fed mice *vs*. chronically alcohol-fed mice and in controls *vs*. alcoholics. Multiple comparisons were performed using two-way ANOVA with Sidak *post hoc* tests for alcohol consumption in a two-bottle drinking paradigm, repeated alcohol withdrawals, and quinine and sucrose intake; Bonferroni *post ho*c test was used for BAC determination. A *p* value of less than 0.05 was considered significant. Analysis was made using GraphPad Prism 7 (GraphPad; La Jolla, CA, USA) and Microsoft Office Excel add-in Analysis ToolPak (Redmond, WA, USA). Outliers were determined using the Outlier function in Excel.

## Results

### Intracellular acidosis in hippocampal neurons by NBCn1 loss

**Figure 1:**
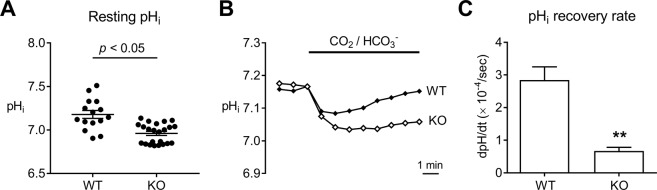
Hippocampal neurons in NBCn1 KO mice develop intracellular acidosis. (**A**) Resting pH_i_. Resting pH_i_ was determined after steady-state pH_i_ was established in the presence of 5% CO_2_, 33 mM HCO_3_^−^ (*n* = 15 neurons from 8 WT postnatal mice and 23 neurons from 11 KO postnatal mice). (**B**) Comparison of pH_i_ recovery from a CO_2_-induced acid load in WT neurons and KO neurons. Recovery of pH_i_ was measured in a CO_2_/HCO_3_^−^-buffered solution containing 10 μM of ethyl isopropyl amiloride to inhibit endogenous Na^+^/H^+^ exchangers. (**C**) Rate of pH_i_ recovery (dpH/dt). Values of dpH/dt (×10^−4^ pH_i_/sec) were measured during the first 2 min after CO_2_-induced acidification (*n* = 31 WT neurons and 28 KO neurons). **p < 0.01 compared to WT neurons.

NBCn1 regulates pH_i_ in neurons and its loss is expected to alter steady-state resting pH_i_ and pH recovery from intracellular acidification. To confirm such changes, we compared resting pH_i_ in hippocampal neuronal cultures between KO mice and WT littermates using the pH fluorescence dye BCECF. Hippocampal neurons were chosen because they play essential roles in mediating drug-related memories^35^. As shown in Fig. 1A, the resting pH_i_ in NBCn1 KO neurons was lower than that in WT neurons (*p* < 0.05, Student t-test; *n* = 23 KO neurons and 15 WT neurons). Furthermore, the rate of pH_i_ recovery from an acidification (dpH/dt) was significantly lower in KO neurons (*p* < 0.01, Student t-test, *n* = 28 KO neurons and 31 WT neurons; Fig. 1B,C). The decrease in dpH/dt corresponded to 76%, indicating that NBCn1 is the major HCO_3_^−^-dependent acid extruder in hippocampal neurons. These results indicate that NBCn1 loss leads to intracellular acidosis in neurons.

### Depression of neuronal excitability by NBCn1 loss

**Figure 2:**
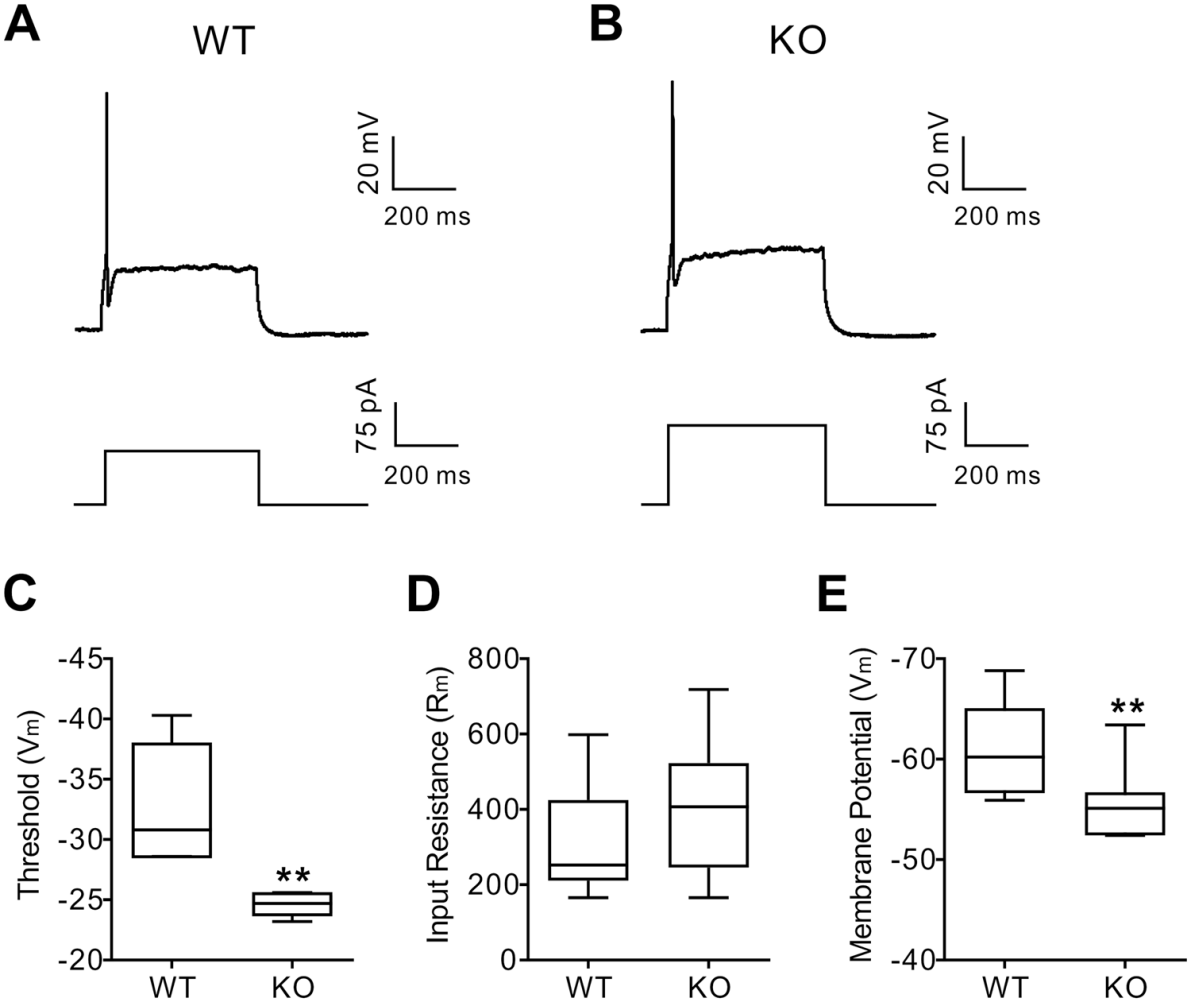
NBCn1 KO neurons have a higher action potential threshold. (**A**,**B**) Representative first action potentials evoked from somatic current injection in WT and KO neurons. Whole-cell recordings in a current clamp configuration were performed to identify the first action potential with a sharp and upward depolarization. (**C**) Action potential threshold. Values were obtained from 9 WT mice and 6 KO mice. **p < 0.01 compared to WT mice. (**D**) Input resistance (R_m_). R_m_ was determined by the slope of a best-fit line in a voltage-current plot. (**E**) Resting membrane potential (V_m_). The membrane voltage in the absence of current injection was determined (*n* = 9 WT mice and 7 KO mice). *p = 0.02 compared to WT.

In our recent report^18^, extracellular recordings from hippocampal slices showed a marked depression of action potentials in NBCn1 KO mice. Because neuronal firing activity is inhibited by acidic pH^36–38^, the depressed action potential in KO neurons could be due to a change in membrane excitability. To test this possibility, we performed patch clamp recordings in a whole cell configuration. In this experiment, intracellular ions are standardized by the patch pipet solution, but pH_i_ is regulated by bath CO_2_/HCO_3_^−^ as CO_2_ is a gas and rapidly penetrates cell membranes. Figure 2A,B show representative first action potentials evoked from somatic current injection in WT and KO neurons. More current was required to produce an action potential in KO neurons than WT neurons. Furthermore, as shown in Fig. 2C, the action potential threshold was more positive in KO neurons (−32.6 ± 1.6 mV, *n* = 9 WT mice *vs*. −24.6 ± 0.9 mV, *n* = 6 KO mice; *p* < 0.01), making it more difficult to excite membranes. KO and WT neurons had similar input resistances (Fig. 2D), indicating that cell size and number of open channels are not different. In addition, the resting membrane potential in KO neurons was more positive (*p* = 0.02; Fig. 2E). Together, these data demonstrate that KO neurons are less excitable.

### Altered alcohol consumption by NBCn1 loss

**Figure 3:**
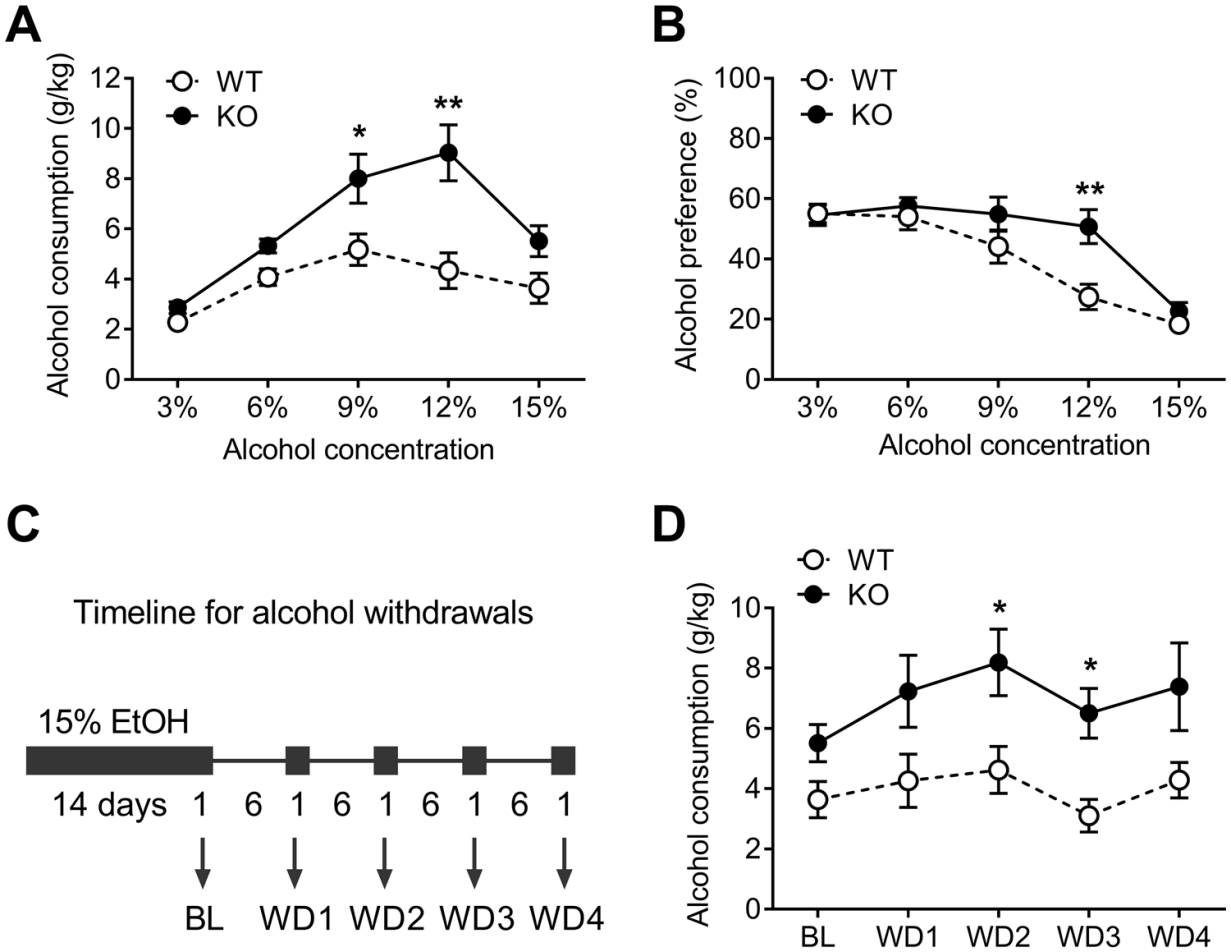
Loss of NBCn1 increases alcohol consumption in mice. NBCn1 KO and WT mice (*n* = 10/group) were allowed free access to water and 3-15% alcohol for 4 days at each concentration, and alcohol consumption (**A**) and alcohol preference (**B**) were measured. Alcohol consumption was calculated as *g* of alcohol per *kg* body weight. Alcohol preference was calculated as percentage of alcohol over total fluid intake (water + alcohol). *p < 0.05 and **p < 0.01 compared to WT mice. (**C**) Schematic timeline for repeated episodes of alcohol withdrawal. Baseline consumption of 15% alcohol was first established in a two-bottle free choice procedure for 14 days and alcohol withdrawal was followed by 4 cycles of 6-day deprivation with 1-day access to alcohol. Alcohol consumption for 24 hrs was measured at the baseline (BL) and after withdrawal (WD1–4). (**D**) Alcohol consumption after repeated withdrawals (*n* = 10/group). *p < 0.05 compared to WT mice.

To assess the effects of NBCn1 loss on alcohol consumption, mice were given continuous access to increasing alcohol concentrations from 3% to 15% (with each concentration available for 4 days before being increased by 3%) and water in a two-bottle choice procedure, and the volumes of alcohol and water consumed were measured. This range was chosen because alcohol consumption in mice proportionally increases until 15%^25^. As shown in Fig. 3A, NBCn1 KO mice consumed more alcohol (*F*_4,72_ = 5.47, two-way ANOVA, *p* < 0.01 for genotype × alcohol percentage interaction; *n* = 10/group). *Post hoc* tests revealed significantly higher alcohol consumption at the 9–15% alcohol range in KO mice. The difference was small at 15% alcohol due to a lower preference for alcohol at high concentrations in C57BL/6 background^39^. The preference for alcohol in KO mice was significantly higher at 12% alcohol than WT mice (*p* < 0.01; Fig. 3B). In separate experiments, mice were first allowed access to 15% alcohol and water in a two-bottle choice until a stable baseline was reached, and this was followed by 4 cycles of 6-day deprivation with 1-day access to alcohol and water (Fig. 3C). In this condition, KO mice consistently consumed more alcohol (*F*_1,18_ = 7. 07, two-way ANOVA, *p* < 0.05; *n* = 10/group; Fig. 3D). The difference between groups appeared to progressively increase over time and reach a steady state. Alcohol consumption in WT mice was not significantly increased after repeated withdrawals, which is somewhat inconsistent with the existing literature^26,40^. The cause of this discrepancy could be due to a relatively short duration of alcohol access prior to the initiation of deprivation cycles in our experiments.

### Negligible change in quinine and sucrose sensitivity by NBCn1 loss

**Figure 4:**
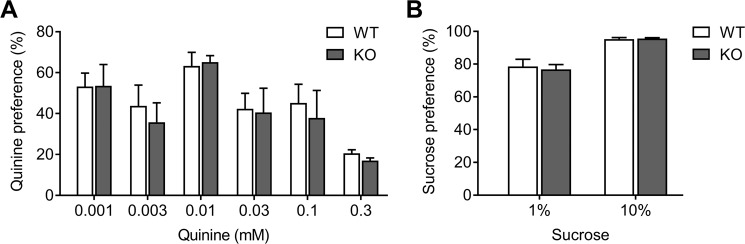
Loss of NBCn1 has no effect on quinine and sucrose sensitivity (**A**) Quinine sensitivity. Mice were offered 0.001–0.3 mM quinine hydrochloride in a two-bottle paradigm and intake was measured. Each concentration was offered for 1 day, with water only for 2 days in between each quinine test day (*n* = 6/group). (**B**) Sucrose sensitivity. Mice were offered 1% or 10% (w/v) sucrose and water for 2 days in a two-bottle paradigm and intake was measured (*n* = 4/group). Quinine and sucrose preference was determined as a ratio of taste solution to total fluid intake.

After baseline intake at the 15% alcohol was established, mice were given access to 0.001–0.3 mM quinine or water in a two-bottle choice, and quinine consumption was measured. No significant effects of genotype (*F*_1,36_ = 0.02, *p* > 0.05) nor genotype × concentration interaction (*F*_1,36_ = 0.01, *p* > 0.05) on quinine preference were observed (Fig. 4A). In separate experiments, a different cohort of mice was given access to 1% and 10% sucrose or water in a two-bottle choice to determine sucrose consumption. No significant effects of genotype (*F*_1,12_ = 0.07, *p* > 0.05) nor genotype × concentration interaction (*F*_1,12_ = 0.13, *p* > 0.05) on sucrose preference were observed (Fig. 4B). Thus, a possible confound of altered taste sensitivity is unlikely involved in altered alcohol consumption in NBCn1 KO mice.

### Increased reward potency of alcohol by NBCn1 loss

**Figure 5:**
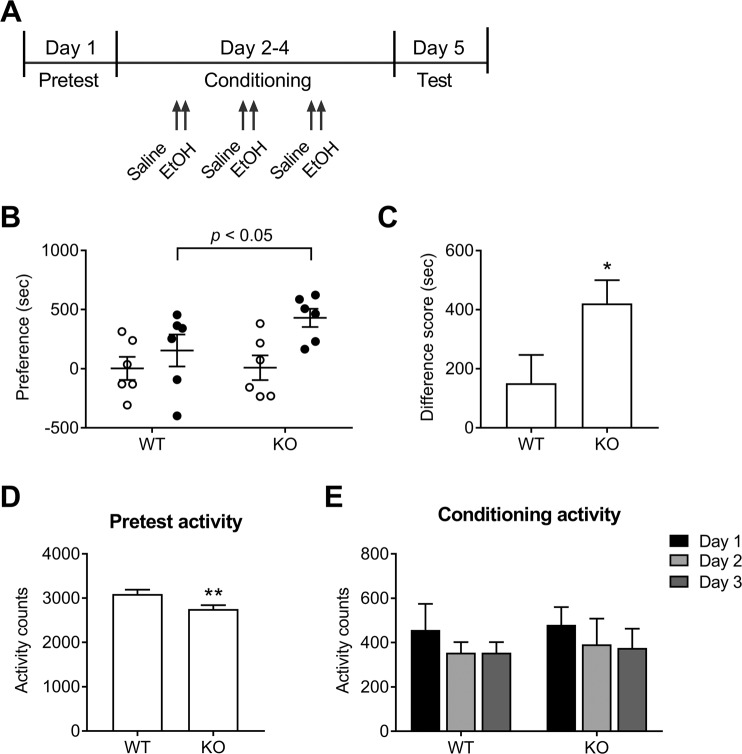
Loss of NBCn1 increases rewarding value of alcohol. (**A**) Timeline of experimental treatment and behavioral measurements in alcohol-induced conditioned place preference (CPP). Following pretest, 5-min conditioning sessions were conducted daily for 3 days, with saline injections in the morning and 2 g/kg of alcohol injections 4 hours later. The test session was conducted by placing mice into the chamber without pretreatment and allowing them to roam the chamber for 15 min. (**B**) Preference scores in pretest (open dots) and test sessions (closed dots). Scores were calculated by subtracting the amount of time spent on the saline paired side from the amount of time spent on the alcohol paired side (*n* = 6/group). (**C**) Difference scores. Difference score is the change in preference scores from pretest to test. *p < 0.05 compared to WT mice. (**D**) Locomotor activity in the pretest session. Locomotor activity was assessed as horizontal activity counts during the 15-min pretest in the compartments equipped with photocell beams. **p = 0.027 compared to WT mice. (**E**) Daily locomotor activity counts during 5-min conditioning. Activity counts were from mice injected with alcohol for 3 consecutive days.

To assess the rewarding value of alcohol, mice were conditioned with 2 g/kg of alcohol (*n* = 6) and preference for the alcohol paired side was determined (Fig. 5A). Control mice received saline on both sides of the CPP apparatus (*n* = 6). KO mice showed increased place preference relative to WT controls (*p* < 0.05; two-way ANOVA; *n* = 6/group; Fig. 5B). This difference was evident when the change in preference score from pretest to test was calculated (Fig. 5C). KO mice had a higher difference score than WT controls. KO mice showed reduced locomotor activity during the pretest session (*p* = 0.02; Fig. 5D), which is essentially an assessment of novel environment-induced exploration within a 15-min period. The activity was, however, similar in the conditioning sessions (Fig. 5E), indicating that the locomotor activity does not differ between groups after alcohol administration.

### Increased sensitivity to alcohol-induced sedation and development of tolerance to sedation by NBCn1 loss

**Figure 6:**
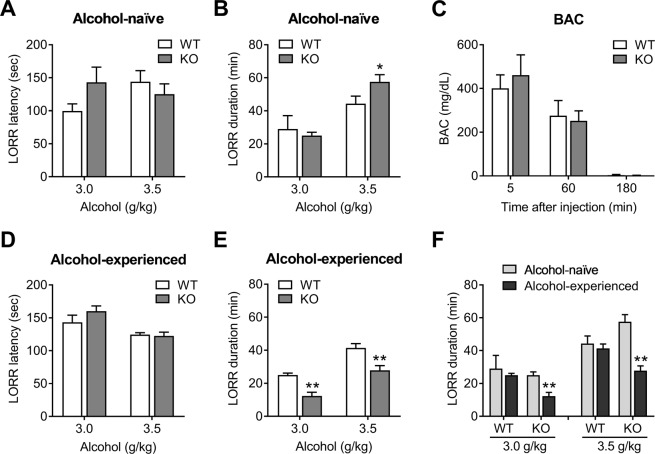
Loss of NBCn1 increases sensitivity to alcohol-induced sedation and induces the development of tolerance to the sensitivity. (**A**,**B**) Loss of righting reflex (LORR) in alcohol-naïve mice. Mice were intraperitoneally injected with 3.0 (*n* = 4/group) or 3.5 g/kg alcohol (*n* = 10/group) and subjected to LORR. LORR latency (**A**) is the time elapsed between alcohol injection and onset of sedation, and LORR duration (**B**) is the time elapsed between onset of sedation and recovery from LORR. Recovery was considered when mice regained an ability to place all four limbs on a V-shape trough twice within a 30-second period. *p < 0.05 compared to WT mice. (**C**) Blood alcohol concentration (BAC) after alcohol injection. Blood samples were collected from the submandibular vein at the indicated time-points after an intraperitoneal injection of 2.0 g/kg alcohol (*n* = 6 WT & 5 KO mice). (**D,E**) LORR in alcohol-experienced mice. Mice were daily injected with 2.0 g/kg alcohol for 7 days and then 3.0 or 3.5 g/kg of alcohol on day 8 (*n* = 4–6/group). Mice were then subjected to LORR. Latency (**D**) and duration (**E**) are described as above. **p < 0.01 com*p*ared to WT mice. (**F**) Comparison of LORR duration between alcohol-naïve *vs*. alcohol-experienced mice. Durations in **B** and **E** were compared to display the effects of alcohol pretreatment on the sensitivity to alcohol-induced sedation in KO *vs*. WT mice. **p < 0.01 compared to WT mice.

A sensitivity to alcohol-induced sedative effects was determined by LORR in which mice were intraperitoneally injected with 3.0 or 3.5 g/kg of alcohol and animals’ inability to right themselves was assessed. These concentrations were chosen to ensure sedation, which is reported to occur at >2.5 g/kg of alcohol^41^. Both groups of WT and KO mice had similar latency to onset of LORR (Fig. 6A), but KO mice had a longer LORR duration at 3.5 g/kg of alcohol (44.3 ± 4.6 min for WT mice *vs*. 57.6 ± 4.3 min for KO mice; *p* = 0.048; *n* = 10; Fig. 6B). Both groups displayed similar blood alcohol concentrations following an intraperitoneal injection of 2 g/kg of alcohol (*n* = 6 WT and 5 KO; Fig. 6C). In another set of experiments, a different cohort of mice was injected with 2.0 g/kg of alcohol for 7 days and then the hypnotic 3.0 or 3.5 g/kg of alcohol was assessed on day 8 (*n* = 4–6/group). This alcohol pretreatment did not change LORR latency (Fig. 6D) but decreased LORR duration in KO mice at both alcohol concentrations (*p* < 0.01 for each; Fig. 6E). The decreased duration (i.e., shorter sleep time) corresponds to a decreased sensitivity to the sedative effects of alcohol in alcohol-experienced KO mice. Figure 6F is a comparison of LORR between alcohol-naïve *vs*. alcohol-experienced mice. Alcohol pretreatment had negligible effect in WT mice, it but reduced the LORR duration in KO mice (*F*_1,13_ = 7.19, *p* < 0.05 for 3.0 g/kg alcohol and *F*_1,28_ = 9.41, *p* < 0.05 for 3.5 g/kg alcohol). Thus, alcohol-experienced KO mice developed tolerance to the sedative effects of alcohol.

### Decreased expression of NBCn1 in the hippocampus and striatum of mice following chronic alcohol consumption

**Figure 7:**
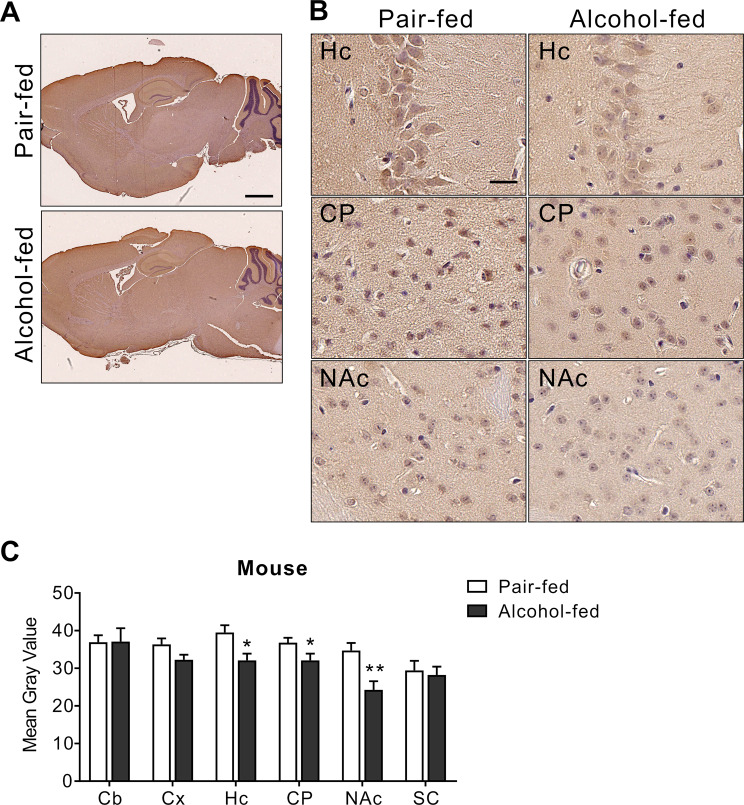
NBCn1 is downregulated in the brains of mice after chronic alcohol consumption. (**A**) Comparison of NBCn1 expression in the brains of pair-fed *vs*. alcohol-fed mice. C57BL/6 mice were fed with 20% alcohol or water in the two bottle free choice procedure for 6 weeks and NBCn1 immunostaining was compared (*n* = 4 mice/group). The scale bar in the upper image is 1 mm and applies to the bottom image. (**B**) Comparison of NBCn1 immunostaining in the pyramidal CA3 region (Hc), caudate putamen (CP), and nucleus accumbens (NAc). The regions were identified using the Allen Brain Atlas (https://mouse.brain-map.org/static/atlas). The scale bar in the upper left image is 20 μm and applies to all images. (**C**) Quantitation of NBCn1 immunostaining. NBCn1-immunostained neurons were randomly selected and quantitated using ImageJ software (n = 10/region). Cb, cerebellum; Cx, cortex; Hc, hippocampus; CP, caudate putamen; NAc, nucleus accumbens; SC, superior colliculus.

The above data from KO mice support the importance of NBCn1 for alcohol-related behaviors, but there is a limitation to understanding how NBCn1 dysfunction and subsequent intracellular acidosis in neurons are associated with alcohol consumption. Studies show that alcohol changes pH_i_ in several peripheral cells^42–44^ and this change is mediated by downregulation of acid-extruding proteins including NBCn1. These reports led us to the hypothesis that alcohol consumption downregulates NBCn1 in neurons. To test this hypothesis, we examined NBCn1 expression levels in the mouse brains following chronic alcohol consumption by allowing C57BL/6J mice free access to 20% alcohol or water (*n* = 5/group) in two-bottle drinking procedures for 6 weeks^45–47^ and then subjecting mouse brains to immunohistochemistry with an NBCn1 antibody^13^. This alcohol feeding method is known to achieve blood alcohol levels of 0.08%^48,49^. NBCn1 immunostaining was decreased in the brains of alcohol-fed mice (Fig. 7A,B). The decrease was particularly evident in the hippocampal CA3 neurons (Fig. 7B, Hc) and also dentate gyrus granular layer (not shown). Negative controls without the primary antibody showed no staining (Supplementary Figure S1). A decrease in NBCn1 was also observed in the striatum, particularly caudate putamen and nucleus accumbens (Fig. 7B, CP & NAc). Quantitative analysis of NBCn1 immunostaining revealed 13–30% decrease in the hippocampus, caudate putamen, and nucleus accumbens (*p* < 0.05 for each, Student t-test; Fig. 7C). Other regions including the cerebellum, cortex, and midbrain (superior colliculus) showed negligible change.

### Decreased expression of NBCn1 in the hippocampus dentate gyrus and dorsal striatum putamen of humans with AUD

**Figure 8:**
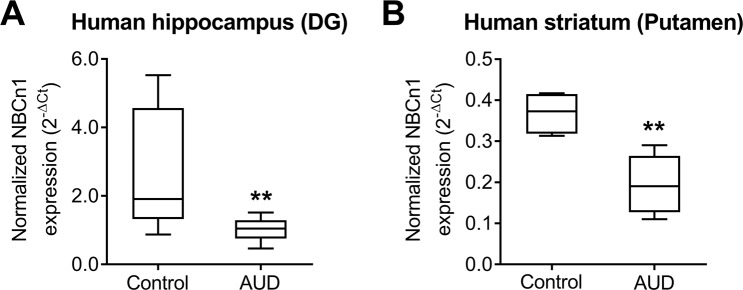
NBCn1 is downregulated in the hippocampus and dorsal striatum putamen of humans with alcohol use disorder (AUD). qPCR was performed on postmortem tissues from individuals with AUD and control individuals (demographic data available in Supplementary Table S1). Experiments were carried out with 10 samples/group, from which 5 samples were randomly selected for dentate gyrus (**A**) and the remaining 5 samples for putamen (**B**). NBCn1 expression was normalized to a geometric mean of references β-actin, UBC1 and TBP. Data were presented as box plot with median and whiskers. **p < 0.01 compared to controls.

To determine whether the above change is not restricted only to mice but can also similarly occur in humans, we examined NBCn1 levels in the hippocampus and dorsal striatum of humans with AUD by qPCR. Postmortem tissues from individuals with AUD and age-matched controls were obtained from the New South Wales Tissue Resource Center^31^ (the demographic data and additional parameters such as PMI, brain pH and RNA quality indicator are listed in Supplementary Tables S1 and S2). Normalized to a geometric mean of three references, β-actin, TBP and UBC1^33,34^, NBCn1 mRNA levels were significantly decreased in the postmortem hippocampus dentate gyrus of humans with AUD (*p* < 0.01; Student t-test, *n* = 5/group; Fig. 8A). A decrease was also observed in the putamen from a separate postmortem cohort of humans with AUD (*p* < 0.05, *n* = 5; Fig. 8B). These results indicate that, similar to the case in mice, chronic alcohol consumption causes NBCn1 downregulation in the hippocampus and striatum of humans.

## Discussion

From this study, we found that NBCn1 loss results in increased voluntary alcohol consumption and increased reward potency of alcohol in mice. NBCn1 loss also results in increased sensitivity to alcohol-induced sedation in alcohol-naïve mice and increased tolerance to the sedation in alcohol-experienced mice. These changes are due to intracellular acidosis and subsequent depression of neuronal excitability. In control mice, NBCn1 is downregulated in the hippocampus and striatum following chronic alcohol consumption, and a similar downregulation occurs in humans with AUD. Because NBCn1 is the primary acid-extruding transporter in neurons, its downregulation is expected to significantly affect pH_i_, comparable to acidification in the knockouts. Thus, chronic alcohol consumption promotes mice to consume more alcohol by reducing NBCn1 expression in a positive feedback mechanism. To our knowledge, our study is the first demonstration that pH contributes to alcohol effects on the reward process and NBCn1 is the key factor for this contribution.

NBCn1 KO neurons show a marked decrease in pH_i_ recovery rate (Fig. 1). Approximately three fourths of the dpH/dt were abolished, indicating that NBCn1 is the major transporter in these neurons. The remaining recovery is likely mediated by other Na/HCO_3_ transporters NCBE and NDCBE, which have been traditionally known to govern HCO_3_^−^-dependent acid extrusion in neurons^5^. A low resting pH_i_ in NBCn1 KO neurons also supports the conclusion that NBCn1 serves as the major transporter in these cells. NBCn1 KO neurons have a higher action potential threshold (Fig. 2), indicating that NBCn1 loss and subsequent acidification depress membrane excitability. This depression is consistent with our recent report that the frequency and amplitude of action potentials in KO hippocampal slices are markedly low^18^. The depressed excitability in NBCn1 KO neurons is also comparable to the characteristics of NCBE (Slc4a10) KO mice[^50^] and NDCBE (Slc4a8)[^51^]. NCBE KO mice show a significant decrease in the frequency of interictal-like events during a pH_i_ recovery from intracellular acidification in the hippocampal CA3 area^50^. Similarly, NDCBE KO mice show decreased population spike amplitude and increased paired-pulse facilitation in the CA1 neurons^51^. These mice have impaired glutamate release from the presynaptic vesicles and decreased frequency of miniature EPSCs, but maintain normal membrane properties including resting membrane potential, action potential threshold, and input resistance. These are different from our observation of a more positive resting membrane potential in NBCn1 KO mice, reflecting a change in ion concentration gradients across cell membranes. NBCn1 can influence ouabain-sensitive Na/K-ATPase^52^ and it is thus possible that NBCn1 loss impairs the pump activity; as a result, K^+^ equilibrium could be shifted to produce membrane depolarization.

KO mice show increased alcohol consumption and alcohol preference (Fig. 3). This difference is not due to altered taste sensitivity, as quinine and sucrose consumption were similar between genotypes. Instead, we think the difference is due to a change in the rewarding value of alcohol. Increased voluntary alcohol consumption can be driven by either decreased rewarding value of alcohol, thus requiring more alcohol to achieve the same level of hedonic response, or increased rewarding value of alcohol, thus leading to enhanced motivation for alcohol consumption^53,54^. In our CPP test, KO mice spent more time in the alcohol-paired compartment than the saline-paired compartment in our CPP paradigm (Fig. 5). Thus, the increased voluntary alcohol consumption in KO mice is driven by an increased rewarding value of alcohol. Nonetheless, we do not exclude a possibility that KO mice may have different rewarding values at different doses of alcohol. The reward system that plays a central role in CPP is the mesolimbic dopamine pathway^55^, which originates in the ventral tegmental area (VTA) and terminates in the nucleus accumbens (NAc) of the basal ganglia^54^. Similar to other drugs of abuse, alcohol stimulates dopamine release from the VTA nerve terminals in the NAc, in part by decreasing local GABAergic inhibition in the VTA, resulting in a disinhibition of dopamine neuronal activity^56^. Furthermore, alcohol has biphasic effects on glutamate release from various glutamatergic projections (including those from the prefrontal cortex, hippocampus, and amygdala) that feed into the VTA and NAc^54^. GABA receptors and NMDA receptors are sensitive to pH^3^, suggesting that a small pH change affects synaptic activity in the VTA and/or NAc neurons. Given a coordination between NBCn1 and NMDA receptors^18^, it is possible that NBCn1 impacts glutamate-mediated alcohol tolerance, dependence, withdrawal, and relapse^57^.

Another interesting finding from the study is an increased sensitivity to the acute sedative effects of alcohol and development of tolerance after a 7-day alcohol administration in KO mice (Fig. 6). We did not observe tolerance development in WT control mice as alcohol-naïve and alcohol-experienced WT mice had similar LORR durations. BACs were similar between genotypes, indicating that tolerance is not due to a difference in alcohol metabolism. We measured BACs with injection of 2.0 g/kg alcohol which equals to the amount of alcohol injected to alcohol-experienced mice in LORR and CPP; nonetheless, we do not exclude a possibility that the two groups of mice could still differ in alcohol metabolism with a 3.0 or 3.5 g/kg dose. In addition, BAC may not be a reliable measure unless both blood and brain alcohol uptake kinetics are studied. Tolerance develops when the effect of a given dose of alcohol decreases with repeated alcohol administration and more alcohol is required to achieve the same effect. In this regard, the tolerance developed in KO mice agrees with increased alcohol consumption. It is interesting to note that KO mice develop a tolerance in relatively short repeats of alcohol administration. Alcohol tolerance can be categorized into three different types based on its development in a timeframe^58^: acute functional tolerance occurring minutes within a single drinking session, rapid tolerance occurring 8–24 hrs after a single alcohol administration, and chronic tolerance lasting for days to years after repeated alcohol administration. The tolerance developed in KO mice is thus considered a chronic tolerance although it occurs after relatively short repeats of alcohol administration. Many molecular mechanisms underlie the development of tolerance, and Pietrzykowski *et al*.^58^ summarizes that mechanisms downstream of protein synthesis are involved in acute tolerance, whereas mechanisms upstream of protein synthesis contribute to chronic tolerance. Thus, NBCn1 loss may change a cellular process upstream of protein synthesis leading to the development of chronic tolerance. The mechanism is presently unclear.

We found NBCn1 downregulation in the hippocampus and striatum after chronic alcohol consumption (Figs. 7 and 8). This downregulation is in line with alcohol-induced decrease in NBCn1 expression in aorta smooth muscles^42^ and oral epidermoid carcinoma cells^43^. Acute alcohol exposure to these peripheral cells results in overall intracellular alkalosis in the presence of CO_2_/HCO_3_^−^, thus reducing acid extrusion. Because NBCn1 is an acid extruder, its decrease after alcohol exposure is conceivable. However, this decrease will likely have variable impact on pH_i_ regulation as these cells express other acid extruders such as V-ATPase, Na/H exchangers, and NBCe1, in addition to NBCn1^42–44^. In contrast, in neurons where NBCn1 is the major acid extruder, NBCn1 downregulation will significantly cause intracellular acidification and subsequently depress neuronal excitability, as shown in KO neurons. We note that the reduced excitability by NBCn1 loss is comparable to the depressant effects of alcohol on neurons. Alcohol depresses membrane excitability and synaptic plasticity^59–61^. In the hippocampus and striatum, alcohol affects memory modulation and influences cognitive and spatial memory and/or stimulus-response habit memories^19,35^. People who are diagnosed with AUD or repeatedly binge drink experience cognitive dysfunction^62,63^. It is thus possible that the reduced excitability by NBCn1 downregulation after chronic alcohol consumption potentiates alcohol’s depressant effects in these memory-associated brain regions. In addition, metabolic acidosis caused by alcohol metabolism may further potentiate alcohol’s depressant effects. Thus, intracellular acidosis by NBCn1 downregulation and systemic acidosis following chronic alcohol consumption will likely serve as a positive feedback process, stimulating a desire to drink while depressing cognitive and spatial memories

There are several limitations in our study. First, all experiments were performed with male mice to minimize a potential sex difference. It is important to state that the results can only be interpreted toward males; the conclusion cannot be applied toward a sex difference. Second, different experiments were conducted to examine various aspects of alcohol-related behaviors in NBCn1 KO mice, and those experiments required varying dosages of alcohol. Thus, there is a limit to accurately ascertain sensitivity to alcohol. Third, we used a single dose of alcohol in CPP and cannot determine whether the dose used is on the ascending or descending limb of the dose-response relationship. And also, we used a CPP apparatus with two compartments. We have reliably observed a dose-dependent alcohol place preference using this apparatus in previous studies^27^, but we also acknowledge that animals’ assignment to preferred or non-preferred compartments can affect the acquisition of CPP^55,64,65^. A three-chambered apparatus with a neutral compartment might be useful to prevent a potential post-condition preference caused by force choice.

In summary, our study shows the importance of NBCn1 for alcohol consumption and sensitivity to alcohol-induced sedation. Given that a small pH difference has been considered minor in function and is often ignored, our finding of a marked change in alcohol-related behaviors by NBCn1 loss is interesting. Our study suggests that acidosis after chronic alcohol consumption potentiates alcohol effects and causes mice to seek more alcohol. The data open up the possibility that inhibiting NBC downregulation has the potential to improve alcohol-related behaviors.

## Supplementary information

Supplementary Information.
