# Supplementary material for: Increased Alcohol Consumption in Mice Lacking Sodium Bicarbonate Transporter NBCn1
Source: Sci Rep. 2020 Jul 3;10:11017. doi: 10.1038/s41598-020-67291-0 (PMC7335059; doi:10.1038/s41598-020-67291-0)
Supplement: Supplementary file 1 — Supplementary Information. [file 41598_2020_67291_MOESM1_ESM.pdf]

Increased Alcohol Consumption in Mice Lacking Sodium Bicarbonate Transporter NBCn1

Jesse R. Schank[1], Soojung Lee[2], Carlos E. Gonzalez-Islas[2], Sadie E. Nennig[1], Hannah D. Fulenwider[1], Jianjun Chang[2], Jun Ming Li[2], Yejin Kim[2], Lauren A. Jeffers[3], Jaegwon Chung[1], Jae-Kyung Lee[1], Zhe Jin[4], Christian Aalkjaer[5], Ebbe Boedtkjer[5], and Inyeong Choi[2,*]

[1]Department of Physiology and Pharmacology, University of Georgia College of Veterinary Medicine, Athens, GA 30602, USA

[2]Department of Physiology, Emory University School of Medicine, Atlanta, GA 30322, USA

[3]Department of Medicine, Pulmonary Division, Emory University School of Medicine, Atlanta, GA 30322, USA

[4]Department of Neuroscience, Uppsala University, Uppsala 75124, Sweden

[5]Department of Biomedicine, Aarhus University, 8000 Aarhus C, Denmark

[*]Correspondence: Inyeong Choi, Ph.D., Department of Physiology, Emory University School of Medicine, Atlanta, GA 30322, USA. Phone 404-712-2092; Fax 404-727-2648; Email: ichoi@emory.edu

Table S1. Demographic data of controls and alcoholics

Subject No.	Age (Years)	PMI (Hours)	Brain pH	Smoking History	RQI	Ethnicity	Liver path	Cause of death	History of psychiatric illness
CONTROLS									
3	63	72	6.9	Ex-smoker	8.1	Caucasian	Congestion	Cardiac: Severe coronary artery atherosclerosis	None
5	43	66	6.2	Not available	6.0	Caucasian	Congestion	Respiratory: Aspiration pneumonia. Complicating multi drug toxicity	None
6	69	16	6.6	Yes	8.2	Caucasian	Normal	Cardiac: Atherosclerotic cardiovascular disease	None
8	57	18	6.6	Ex-smoker	7.8	Caucasian	Congestion	Cardiac: Ischemic heart disease	None
11	47	38	6.74	Yes	8.6	Caucasian	Steatosis	Cardiac	None
17	58	28	5.92	Yes	5.7	Caucasian		Cardiac	None
18	59	40	6.53	Ex-smoker	6.4	Caucasian	Normal	Cardiac	None
19	59	43	6.69	Yes	8.4	Caucasian	Steatosis	Cardiac	None
27	69	52	6.95	No	8.6	Caucasian	Normal	Cardiac	None
29	54	29	6.8	No	7.3	Asian	Steatosis	Cardiac	None
ALCOHOLICS									
15	54	17	6.41	Yes	8.3	Caucasian	Steatosis	Trauma: Chest and abdominal injury, Ischemic Heart Disease	None
16	55	17	6.85	No	8.5	Caucasian	Congestion	Respiratory	Depression for 17 years, no antidepressants
17	55	48	7.02	Yes	8.6	Caucasian	Cirrhosis	Cardiac	Depression/anxiety for 6 years, treated with antidepressants
19	58	44.5	6.47	Yes	8.2	Caucasian	Cirrhosis	Cardiac	None
22	60	16.5	6.48	Yes	8.6	Caucasian	Cirrhosis	Hepatic	None
24	63	25.5	6.21	Yes	5.0	Caucasian	Congestion	Cardiac	None
25	64	39	6.76	Yes	9.0	Caucasian	Steatosis	Toxicity	Depression for 3 years, treated with antidepressants.
26	69	22	5.82	Yes	5.7	Caucasian	Congestion	Toxicity	Depression for 50 years, treated with

									antidepressants for 4 years (27 years prior)
27	70	62	6.82	Yes	7.1	Caucasian	Congestion	Cardiac: Cardiomyopathy	Depression and suicidal 12 months prior to death, treated with antidepressants
29	43	29	6.29	Yes	6.2	Caucasian	Steatosis	Blood loss	None

PMI, post-mortem interval; RQI, RNA quality indicator.

Table S2. Sample demographic information.

Characteristics	Controls	Alcoholics	*P* value
Number	10	10	
Age (years)	57.8 ± 2.65	59.1 ± 2.53	0.99
PMI (h)	40.2 ± 5.95	32.1 ± 4.95	0.22
Brain pH	6.6 ± 0.10	6.5 ± 0.11	0.99
RQI	7.5 ± 0.35	7.5 ± 0.45	0.99

Data are shown as mean ± SEM, and the difference between controls and alcoholics was tested with Student t-test.

Table S3. qPCR primers

Gene	Forward Primer	Reverse Primer
NBCn1	GTAATGGAAGTGGTGGAAGCAGAG	GGATCCCACTCTCCTGGAG
UBC1	CGGTGAACGCCGATGATTAT	ATCTGCATTGTCAAGTGACGA
TBP	GAGCTGTGATGTGAAGTTTCC	TCTGGGTTTGATCATTCTGTAG
β-actin	CCTGGCACCCAGCACAAT	GGGCCGGACTCGTCATACT

Data are shown as mean ± SEM, and the difference between controls and alcoholics was tested with Student t-test.

Table S4. Cytotoxicity in primary cultures from mice

**Postnatal**	**# of mice**	**Cell/well**	**LDH release (%)**
P5	3	1×10^4	17.5 ± 1.14
P7	3	1×10^4	16.1 ± 0.43

LDH release: Neurons in a 24-well plate (1×10^4 cells/well) were rinsed twice with cell buffer and treated with 1% Triton X-100 or none. LDH release from neurons was quantitated using the Cytotoxicity Detection kit LDH (Roche). LDH release in the presence of Triton X-100 is the total release. Cytotoxicity was expressed as the percentage of total LDH release after subtracting background (no cells). The results show 83–84% cell survival.

Figure S1.

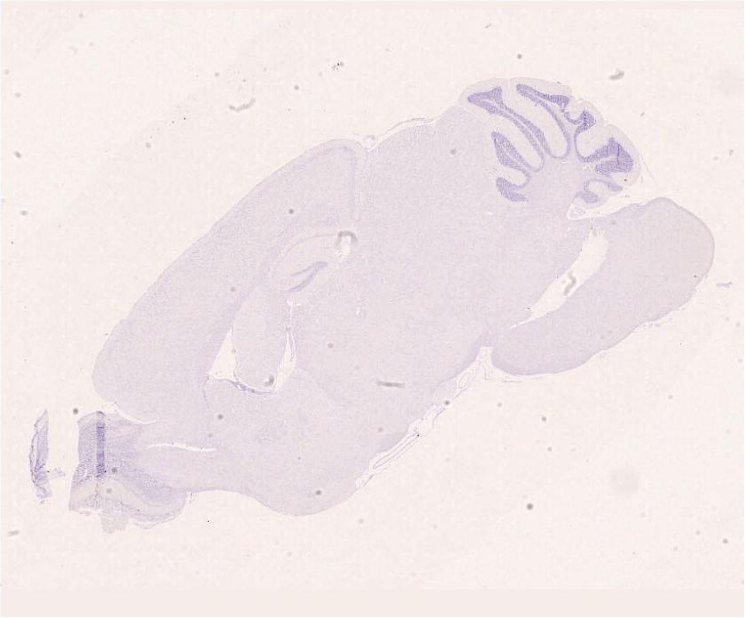

Figure legend. Negative control in immunohistochemistry. Immunostaining without a primary antibody was performed as described in the main text. Briefly, mouse brain tissues were fixed in 4% paraformaldehyde, dehydrated in 30% sucrose, and embedded in paraffin. Paraffin sections (5 μm thick) were heated, deparaffinized in xylene, rehydrated through graded alcohol rinses, and incubated in 3% H_2O_2 for 20 min. Antigen retrieval was done by heating in 10 mM citrate buffer (pH 4.0) in a microwave for 2 min. Sections were blocked with 1% bovine serum albumin in phosphate-buffered saline (PBS) and then with PBS at 4°C overnight. Sections were then incubated with an anti-rabbit horseradish peroxidase-conjugated secondary antibody (cat. #: 7074; Cell Signaling Technology, USA) diluted at 1:1000 for 1 hr at room temperature. Sections were stained with 3,3′-diaminobenzidine (DAB) and then counterstained with hematoxylin.
